# Slip Flow Analysis in an Experimental Chamber Simulating Differential Pumping in an Environmental Scanning Electron Microscope

**DOI:** 10.3390/s22239033

**Published:** 2022-11-22

**Authors:** Pavla Šabacká, Jiří Maxa, Robert Bayer, Petr Vyroubal, Tomáš Binar

**Affiliations:** 1 Institute of Scientific Instruments of the CAS, Královopolská 147, 61200 Brno, Czech Republic; 2 Department of Electrical and Electronic Technology, Brno University of Technology, 61600 Brno, Czech Republic

**Keywords:** Ansys Fluent, slip flow, shear stress, nozzle, low pressure

## Abstract

This paper describes the combination of experimental measurements with mathematical–physical analysis during the investigation of flow in an aperture at low pressures in a prepared experimental chamber. In the first step, experimental measurements of the pressure in the specimen chamber and at its outlet were taken during the pumping of the chamber. This process converted the atmospheric pressure into the operating pressure typical for the current AQUASEM II environmental electron microscope at the ISI of the CAS in Brno. Based on these results, a mathematical–physical model was tuned in the Ansys Fluent system and subsequently used for mathematical–physical analysis in a slip flow regime on a nozzle wall at low pressure. These analyses will be used to fine-tune the experimental chamber. Once the chamber is operational, it will be possible to compare the results obtained from the experimental measurements of the nozzle wall pressure, static pressure, total pressure and temperature from the nozzle axis region in supersonic flow with the results obtained from the mathematical–physical analyses. Based on the above comparative analyses, we will be able to determine the realistic slip flow at the nozzle wall under different conditions at the continuum mechanics boundary.

## 1. Introduction

Currently, research on environmental electron microscopy is being conducted at the Institute of Scientific Instruments of the CAS in Brno in cooperation with the Department of Electrical and Electronic Engineering of the Brno University of Technology.

The environmental scanning electron microscope (ESEM) allows, due to the presence of gas pressures in the order of units of thousands of Pa in the specimen chamber, the observation of electrically non-conductive or semiconductive samples without charge artifacts [1], the observation of sensitive biological [2] and polymeric [3] samples without damage in their native state by special methods [1], or the study of these samples in dynamic in situ experiments [3]. Signal electrons are detected in ESEMs using special ionization [4,5] or scintillation detectors [6,7,8], whose images can be correlated with light microscope images [9].

Recently, an experimental chamber simulating the pumping conditions in an environmental electron microscope was fabricated, where two chambers with a large pressure gradient were separated by a small deferentially pumped region with a Laval nozzle-shaped opening [3,10]. Typically, in practice, a pressure of 2000 Pa is used in the specimen chamber (further denoted as P1), and a pressure of approximately 100 Pa is used in the differentially pumped chamber (further denoted as P2). In its design, simulations were performed to determine the final shape of the Laval nozzle, according to Prandtl’s theory presented in [11].

In this paper, the theory and simulations used in preparing an experiment on this chamber to evaluate the slip flow regime at low pressure conditions are presented.

Throughout the whole experimental chamber system, membrane sensors, appropriated for the expected pressure and its changes, were deployed. Thus, differential BD Sensors DPS 300 were chosen for their high-accuracy measurements and ability to avoid overloading the experiments described later in the article.

## 2. Initial Experiment

**Figure 1:**
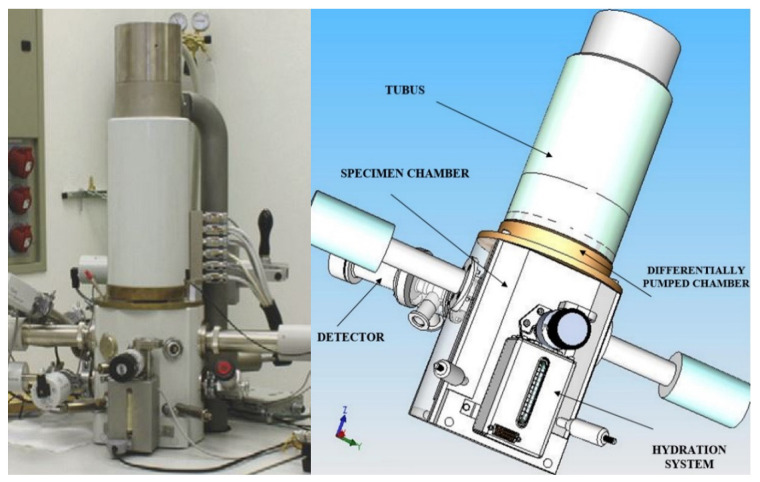
Electron microscope AQUASEM II.

The mathematical–physical analyses discussed in this paper were preceded by experiments in the field of the pumping of the differentially pumped chamber of the environmental scanning electron microscope AQUASEM II (ESEM AQUASEM II), a piece of equipment which was developed at the ISI CAS by the team of Vilém Neděla. This microscope is shown in Figure 1, both in its actual form on the left and in a 3D volume model on the right. It consisted, among other components, of a specimen chamber and a differentially pumped chamber, which were separated by a small aperture. This small aperture, typically 0.5 mm in diameter, ensured that the desired pressure ratio between the specimen chamber and the differentially pumped chamber was maintained during pumping.

**Figure 2:**
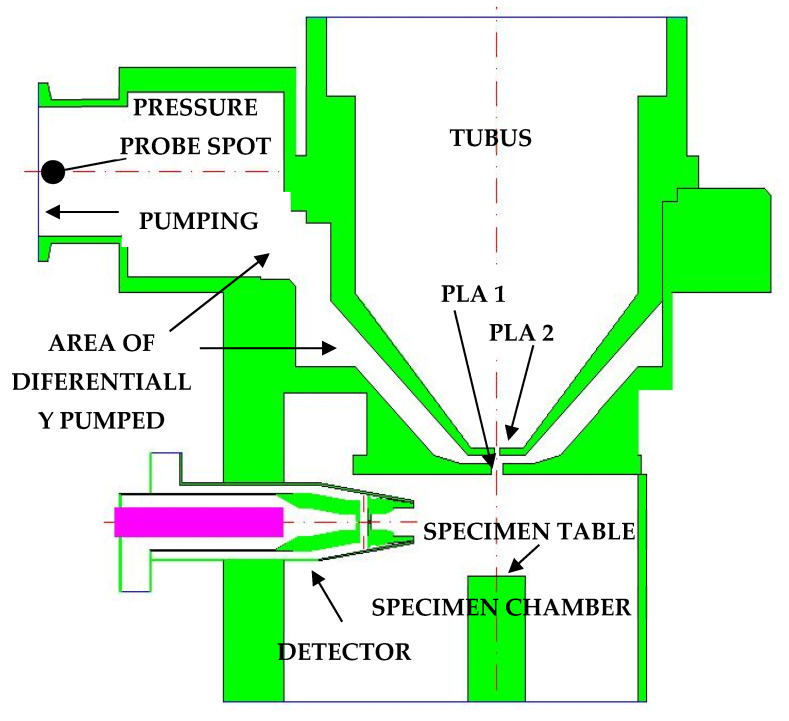
Electron microscope AQUASEM II sectional view with Boundary Condition.

Figure 2 shows a sectional view of the given microscope, focusing on the parts that were relevant to the comparative experiment with the mathematical–physical analyses. These were mainly the specimen chamber, separated by a pressure-limiting aperture PLA 1 of 0.5 mm diameter from the differentially pumped chamber, which was separated from the tubus by a PLA 2 aperture of 0.05 mm diameter. However, to simplify the experiment, the PLA 2 aperture was closed.

In practice, the pumping of a given chamber was performed in two stages. The tubus was pumped in two stages at an operating pressure of 0.01 Pa, and the chamber was differentially pumped using a Lavat RV 100/1 rotary oil pump with a pumping rate of 0.00694 m^3^/s. However, for the experiment, the PLA 2 aperture was blocked and neglected as it did not affect the aim of the whole experiment, and the tubus did not need to be pumped.

Under these conditions, the experimental pumping of ESEM AQUASEM II was carried out at ISI CAS Brno and its results were used to tune the ANSYS Fluent system for the subsequent analyses. Described hereafter, these serve as a basis for the preparation of the other planned experimental measurements mentioned in the article.

For the mathematical and physical analysis, boundary conditions were set according to the real conditions used in the experiment (Figure 2). A closed wall was set at the PLA 2 aperture location and the tubus was excluded from the experiment. At the pumping throat, stretched by the length of the used hose of 1 m, the pumping speed was set to the same value as the pumping power of the used pump.

**Figure 3:**
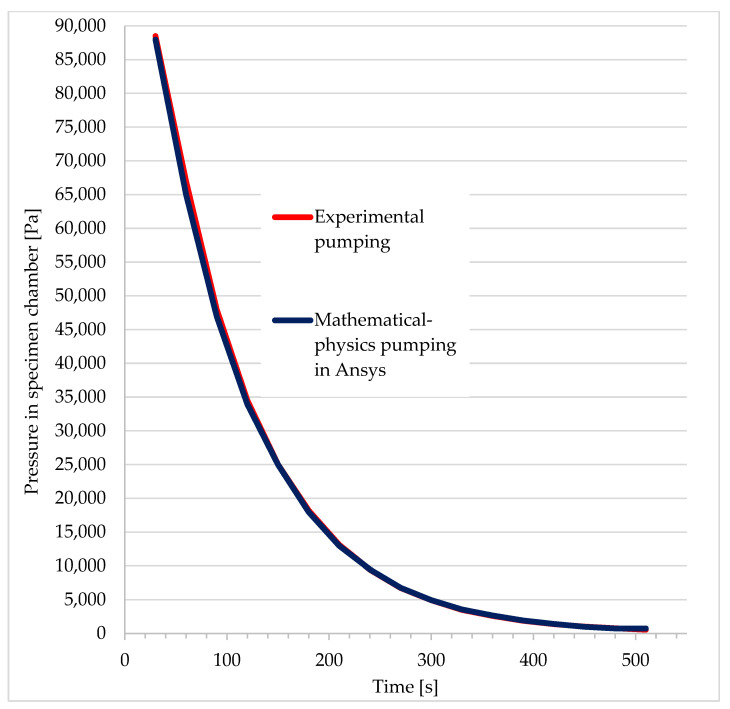
Comparison of experimental results with mathematical and physical analysis.

The results are presented in Figure 3, showing the pressure drop in the specimen chamber during its pumping and comparing it with the results obtained by mathematical and physical analysis. The results are practically identical.

**Figure 4:**
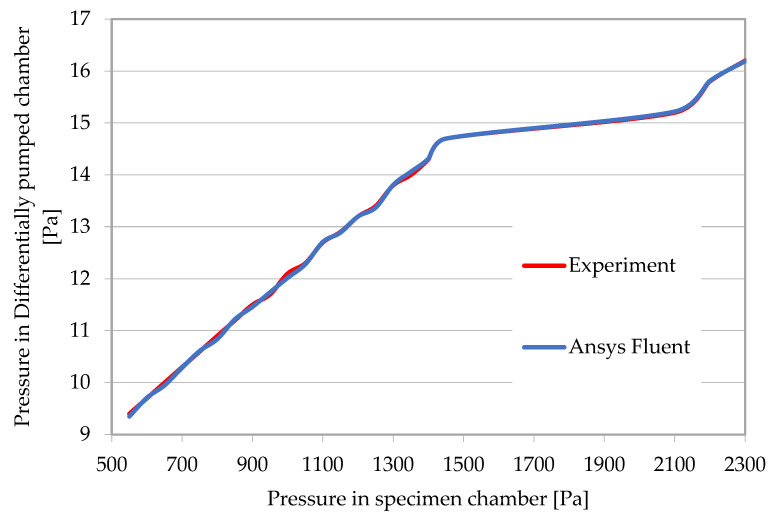
Comparison of differentially pumped chamber pressures obtained from experimental measurements and Ansys Fluent analyses.

At the same time, the results of the experimental measurements were compared with the Ansys Fluent analysis at the control point at the pumping throat (Figure 3). The results are presented in Table 1, and they reveal a very good correlation. The values from Table 1 are further plotted in a graphical dependency (Figure 4), where the correlation between the experiment and the mathematical and physical analyses is more evident.

The results, demonstrating the correspondence between the experiment and mathematical–physical analyses, confirm that the computational setup was able to map the identically challenging nature of the pressure drop flow at the continuum boundary. It also managed to adequately map the physics of the critical flow that arises in the small-sized aperture, separating the specimen chamber from the differentially pumped chamber. Here, the supersonic flow with a reduced low-pressure region is generated, which strongly affects the pumping. Thus, the correspondence of the results demonstrates that the setup matches the necessary conditions for the given physics.

Since the analyses confirmed the assumption of supersonic flow with large gradients, the density-based solver in the ANSYS FLUENT system was chosen. The density-based solver solves the governing equations of continuity, momentum, and (where appropriate) energy and species transport simultaneously as a set, or vector, of equations. Governing equations for additional scalars will be solved sequentially i.e., segregated from one another and from the coupled sets. Two algorithms are available for solving the coupled set of equations, these being the coupled-explicit formulation, and the coupled-implicit formulation.

Two formulations exist under the density-based solver: implicit and explicit. The implicit and explicit density-based formulations differ in the way they linearize the coupled equations. Due to the broader stability characteristics of the implicit formulation, a converged steady-state solution can be obtained much faster using the implicit formulation rather than the explicit formulation. Out of these two formulations, we chose the implicit option due to the complexity of the flow type. In implicit formulation, each equation in the coupled set of governing equations is linearized implicitly with respect to all dependent variables in the set. This will result in a system of linear equations with N equations for each cell in the domain, where N is the number of coupled equations in the set. In summary, the coupled implicit approach solves all variables in all cells at the same time. In view of the above, the mathematical–physical analysis we tuned proved the correctness of the chosen solver with implicit formulation, which is suitable for the complex case of large pressure gradients in the supersonic flow with a significant pressure drop during th pumping of the microscope chambers.

To set the Flux Type, the AUSM scheme was chosen. The AUSM scheme has several desirable properties:

- Provides exact resolution of contact and shock discontinuities
- Preserves positivity of scalar quantities
- Free of oscillations at stationary and moving shocks

In particular, the first point is beneficial for calculations with large gradients.

As a result, the second-order upwind scheme was chosen for discretization. When second-order accuracy is desired, variables at cell faces are computed using a multidimensional linear reconstruction approach [12]. In this approach, higher-order accuracy is achieved at cell faces through a Taylor series expansion of the cell-centered solution about the cell centroid [13].

The settings, given above, fully managed this type of very complex flow and corresponded to the results of experimental measurements. This type was also the basis for the subsequently described mathematical–physical analyses in the area of slip flow, which were also the basis for another experiment. It is constituted by a series of subsequent experiments and mathematical–physical analyses gradually examining conditions on the border of continuum mechanics for use in ESEM.

## 3. Experimental Chamber

**Figure 5:**
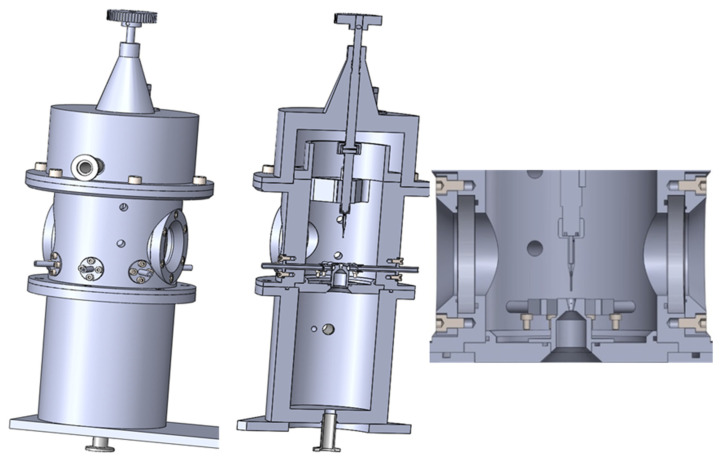
Experimental chamber [11].

Based on existing mathematical–physical analyses, an experimental chamber was designed to study the gas flow at the continuum mechanics boundary. It consists of two chambers separated by an aperture with a diameter of 2 mm, causing critical flow accompanied by supersonic flow inception behind the aperture [14]. The designed chamber is built so that researchers can examine different aperture shapes and place various sensors, as well as a temperature sensor, behind the chamber’s nozzle to capture the static and total pressure in the gas flow (Figure 5). The chamber was lent to the Department of Electrical and Electronic Engineering of the Brno University of Technology by the Institute of Scientific Instruments of the CAS in Brno which manufactured it.

## 4. Ansys Fluent

The Ansys Fluent solves the physics of the flow with the continuum method. Due to the supersonic flow character with large gradients, the density-based solver was set for the calculation with the second-order discretization.

At normal atmospheric pressure, the gas behaves as a continuous medium where various forces between molecules such as gravity, pressure, and friction against adjoining particles come into play, and turbulence is generated based on these forces [15]. As mentioned earlier, Navier–Stokes equations are used to solve the continuous medium, which is derived from the mentioned forces acting on the different parts of the fluid. The fluid state is described by its velocity and pressures at all points where the fluid is located.

When the pressure and therefore the density of the gas decreases, the distance between the molecules increases, the forces cease to act, and the state of the gas is given by the motion of the free molecules.

The Knudsen number can be verified by Equation (1):

(1) $$Kn=\frac{\lambda }{L},$$

where *λ* is the mean free path of the gas molecules and *L* is the characteristic dimension [16].

The mean free path can be further calculated based on (2):

(2) $$\lambda =\frac{kT}{\sqrt{2}\pi \delta^{2}p},$$

where *k* is the Boltzmann constant, *T* is the absolute temperature, *δ* is the diameter of the gas molecule, and *p* is the pressure.

**Figure 6:**
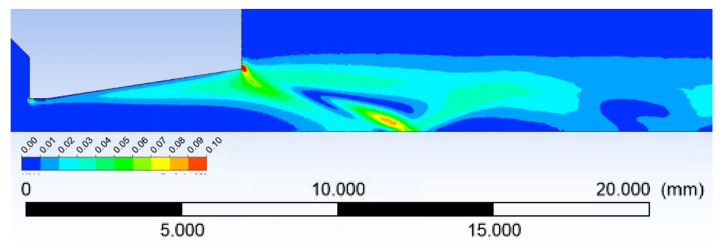
Knudsen number distribution [11].

A method, successfully published by the FOM Institute for Plasma Physics Rijnhuizen and the Department of Applied Physics, Eindhoven University of Technology [17], was used to map the Knudsen number in a dimensionally different space. According to that method, the characteristic dimension L was calculated as a density/density gradient. The distribution of the Knudsen number in the Laval nozzle region is shown in Figure 6.

**Figure 7:**
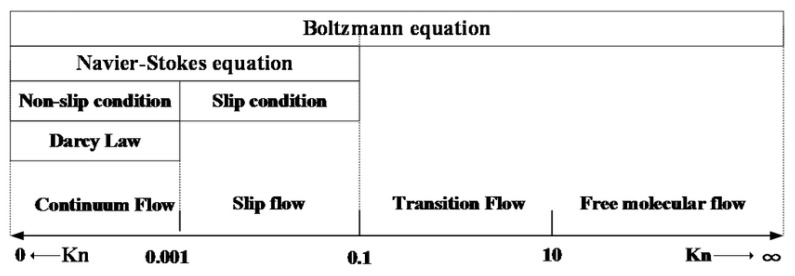
Flow regimes as a function of Knudsen number.

The value of the Knudsen number can be used to determine which type of flow is involved. In practice, as seen in Figure 7, four types of flow are commonly distinguished according to the value of the Knudsen number:

- *Kn* < 0.01      Continuous (viscous) flow
- 0.01 < *Kn* < 0.1   Slip flow
- 0.1 < *Kn* < 0.5  Transient flow
- *Kn* > 0.5      Movement of free molecules

In our case, these are the boundary conditions between no-slip and slip flow.

**Figure 8:**
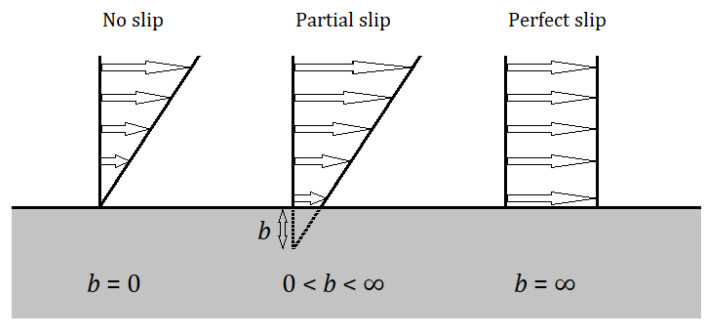
Slip regimes.

In continuous flow, there are no specific or special flow phenomena. In the case of slip flow, there are special phenomena that can be modeled by a specific modified continuum theory according to the wall flow, where there is zero flow and then a so-called slip flow. Slip flow can be further divided into three types (see Figure 8). The transient flow is the type of flow between the slip flow and the movement of free molecules, which has already been analyzed statistically, for example via the Boltzmann equation. In the movement of free molecules, inertial and binding forces no longer play a role, and this type of flow has to be solved completely statistically [18,19].

Figure 8 shows the differences between the different types of slip flow. The no-slip flow at the wall shows zero flow velocity at the wall, and the velocity increases towards the center of the flow. Partial slip flow starts already behind the wall, whereas there is considered to be zero current velocity inside the wall material, and thus there is already some current velocity at the wall and slip flow occurs. In the perfect slip flow variant, the flow velocity is the same in all layers of the flow.

As the Knudsen number increases further into the free molecular flow regime, there is no interaction between the gas molecules approaching the particle surface and those leaving the surface. Therefore, gas molecules arriving at the surface will have full free-flow velocity.

In slip flow, the velocity of the gas phase at the solid surface differs from that of the wall, and the temperature of the gas at the surface differs from that of the wall. Maxwell models are used for these physical phenomena in Ansys Fluent because of their simplicity and efficiency [20].

## 5. Shear Stress Analysis in Experimental Nozzle

The above-mentioned problems were analyzed using mathematical–physical analysis on the model of the prepared experimental chamber.

**Figure 9:**
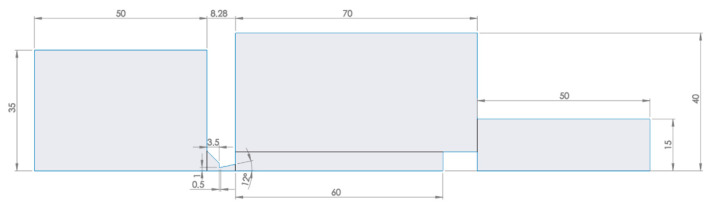
2D model of the experimental chamber.

A 2D model was created for the mathematical–physical analysis and its dimensions are shown in Figure 9.

In the first step, a time-varying calculation was performed in which a given chamber was depressurized from atmospheric pressure to the predicted pressure ratio:

- P1 = 2000 Pa
- P2 = 100 Pa

**Figure 10:**
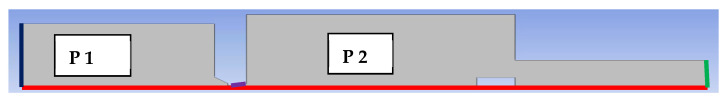
Boundary conditions on the 2D model of the experimental chamber.

The boundary conditions, in this case, are set to:

Inlet to chamber P1 (blue area in Figure 10) at mass flow rate = 0.

The outlet of chamber P2 (green area in Figure 10) has been set to the pumping rate of the planned Lavat pump which was converted to a flow rate of 2 m/s for a given cross-section.

The calculation was solved as axisymmetric, where the axis of symmetry is marked in red in Figure 10.

The time step was set to 0.01 s and the maximum number of iterations was set to 300, which was fully sufficient to run the task. After a few time steps, the minimum number of iterations was sufficient to converge each step.

Air was used as the ideal flow medium with the following properties at the start of the calculation:

Specific Heat:       1006.43 J∙kg^−1^∙K^−1^
Thermal Conductivity:   0.0242 W∙m^−1^∙K^−1^
Viscosity:         1.7894∙10^−5^ kg∙m^−1^∙s^−1^
Molecular Weight:     28.966 kg∙kmol^−1^

For this first calculation, a no-slip mode was set on the chamber walls, including the nozzle. We sought to evaluate the shear stress on the walls during pressure reduction in the chambers, and in particular, in the nozzle itself, marked in purple in Figure 10.

**Figure 11:**
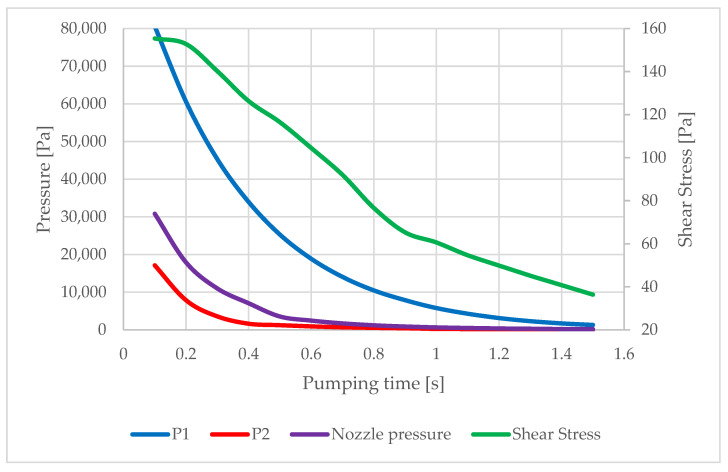
Evolution of monitored variables during chamber pumping.

During the calculation, both the values for convergence checking of the calculation i.e., residuals and monitors of the main variables and the values needed for the actual evaluation of the calculation, namely the pressure drop in chambers P1 and P2 over time, as well as the pressure and shear stress on the nozzle wall, were mapped. The results are shown in Figure 11.

**Figure 12:**
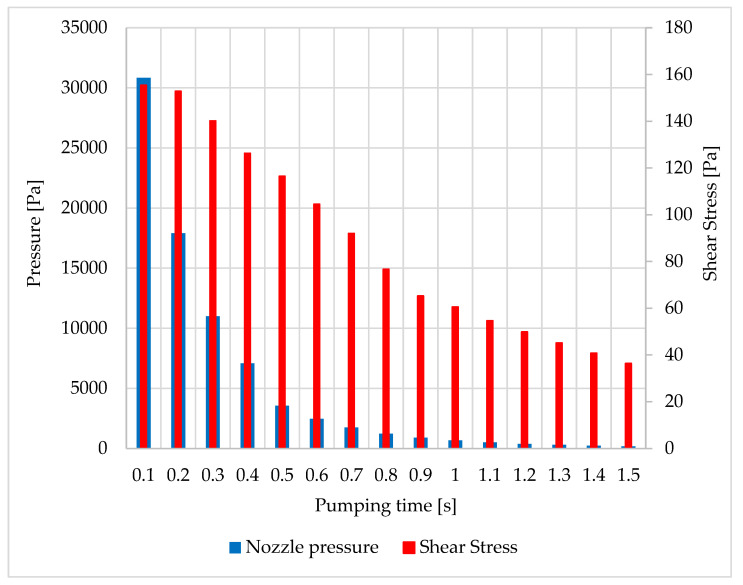
The course of monitored variables during chamber pumping.

Figure 11 shows the predicted pressure drop in the individual chambers and the associated pressure drop in the nozzle. It can be seen from the results that, from a pressure of approximately, 30,000 Pa the wall shear stress starts to decrease. Figure 12 shows the relationship between the average pressure and the average value of the shear stress on the nozzle wall in more detail, showing that this is not a linear relationship.

For further analyses, a variant of the pressure ratios was selected from the time-varying problem that corresponds to the pressure ratios between the chambers for which the whole series of planned experiments will be performed. Namely, the variant chosen occurred at time 1.3 s, when the pressure ratio P1 = 2332.5 Pa and P2 = 118.3 Pa, during pumping in the chambers separated by the aperture, as seen in Table 2. This is a pressure ratio often used in ESEM.

Thus, the time-varying problem becomes the basis for further time-steady analyses, where the boundary conditions correspond to the pressures in both chambers. The analyses presented hereafter are preparatory for the slip flow experiment, but the results will also form the basis for experiments to follow in the short future.

### Evaluation of Slip Flow Variants

For the given conditions, three variants of the mathematical and physical model settings of the experimental chamber were performed to evaluate the nature of the flow at different shear stress settings on the walls, especially at the nozzle, to evaluate the flow regime at the slip flow boundary.

The calculations were performed as steady-state, time-invariant tasks for the selected conditions from the previous time-varying task. The advantage of the time-invariant problem is the possibility of a deeper convergence of a given condition and faster attainment of the results when multiple variations—in this case within the Slip Flow region—are chosen for given pressure ratios. However, it is possible to perform a specific mesh adaptation for a given pressure ratio, as will be discussed below.

**Figure 13:**
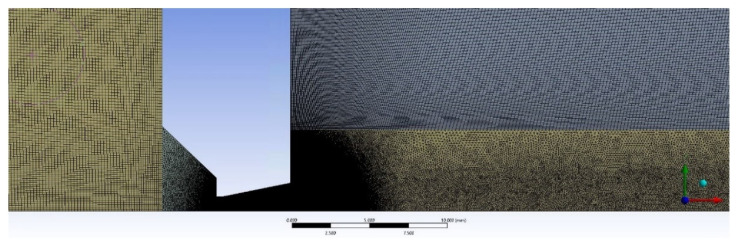
Mesh at the beginning of the calculation.

Figure 13 shows the mesh at the beginning of the calculation. Very significant refinement of the mesh can be seen where the pressure gradients are expected to occur. In that region, the mesh consists of an unstructured cell mesh. In the other regions, which are outside the displayed area, the mesh is structured to save cells.

**Figure 14:**
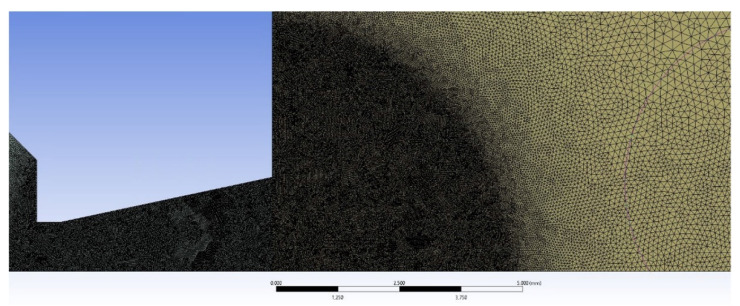
Detailed view of the nozzle area with a distinctly fine mesh.

The following Figure 14 is a close-up view of a nozzle region with a distinctly fine mesh.

**Figure 15:**
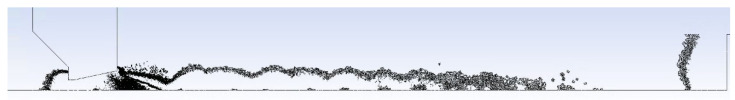
Detailed view of the area of the first shock wave behind the nozzle.

**Figure 16:**
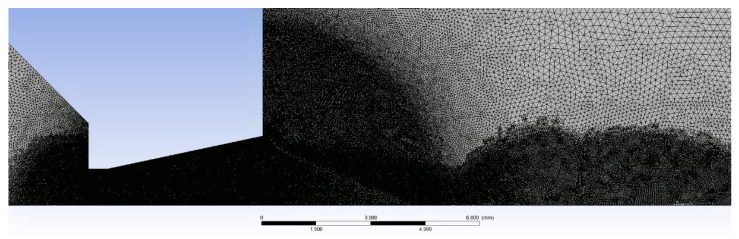
Detailed view of the area of the first shock wave behind the nozzle—zoomed.

Multiple manual adaptive refinements were performed according to the pressure gradient during the calculation. This resulted in refinement in the regions of the shock waves, shown further down. Figure 15 and Figure 16 show a close-up view of the first shock wave region shortly after the nozzle.

Convergence of the calculation was monitored using residuals with convergence requirements:

- Continuity-10^−3^
- X-velocity-10^−3^
- Y-velocity-10^−3^
- Energy-10^−3^

Due to the density-based option, the energy setting is also set to 10^−3^. In practice, however, the calculation was extended to include more iterations, as in addition to monitoring the residuals, the monitors were set to monitor the pressure, temperature, and velocity in the whole volume, as well as pressure in each chamber separately. The calculation was declared finished only after all monitors reached equilibrium.

The following three mode variations were chosen for the calculations:

- For the first variant—No Slip—the walls are set to no-slip mode. This variant was chosen as a reference to compare the nature of the flow with no-slip with subsequent variants with slip.
- For the second variant—Low-Pressure Boundary Slip—slip flow mode is set on the walls in Ansys Fluent, respecting the lower pressure condition using Maxwell’s model [13]:
  (3) $$U_{w}-U_{g}=\left(\frac{2-\alpha_{v}}{\alpha_{v}}\right)K_{n}L_{c}\frac{\partial U}{\partial n}\approx \left(\frac{2-\alpha_{v}}{\alpha_{v}}\right)\frac{\lambda }{\delta }\left(U_{g}-U_{c}\right)$$
  (4) $$V_{g}≣{\left(\vec{V}\vec{n}\right)}_{g}=V_{w}$$
  where *U* and *V* represent velocity components that are parallel and perpendicular to the wall. The indices *g*, *w* and *c* indicate the velocities of the gas, the wall and the center of the cell, respectively. *δ* is the distance from the center of the cell to the wall. *L_c_* is the characteristic length. *α_v_* is the momentum accommodation coefficient of the gas mixture, and its value is calculated as the mass-weighted average of each gas in the system.
  This option was chosen as an evaluation of the solution of a given mathematical Maxwell model for ESEM conditions with the expected effect of a low slip.
- For the third variant—20 Pa version—the nozzle walls are given the shear stress evaluated from the nozzle walls from the first variant, converted to a value of 20 Pa, as the lowest that occurs on the wall at a given ratio.
  This option was chosen to evaluate the transferred data from a time-varying task during which shear stress on the walls during the pumping process was evaluated. The lowest value of shear stress on the nozzle wall was selected from a given time step and transferred to that task.

**Figure 17:**
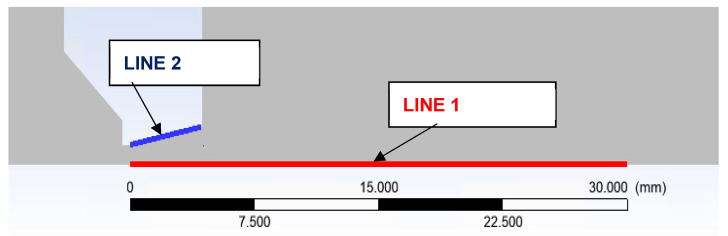
Selected paths for plotting results.

**Figure 18:**
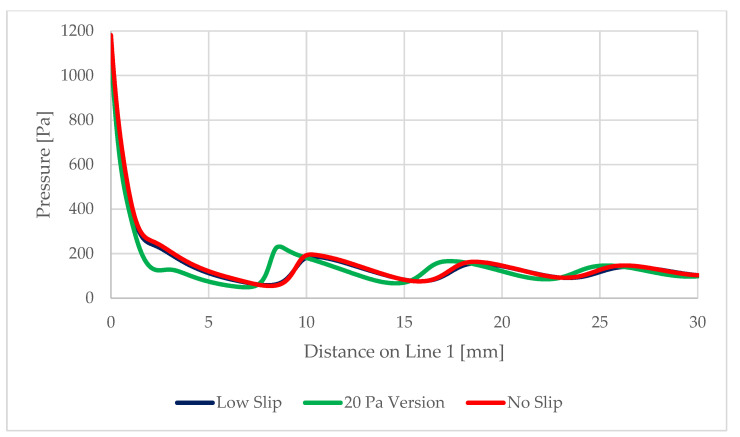
Static pressure distribution on Line 1.

**Figure 19:**
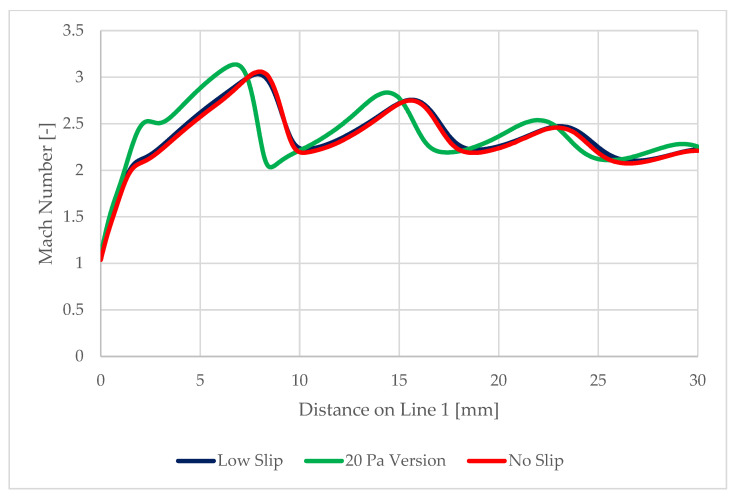
Mach number evolution on Line 1.

On the path of the main flow direction through the nozzle, marked as Line 1 in Figure 17, the following quantities were plotted:

- Static pressure waveform (Figure 18)
- Mach number (Figure 19)
- Static temperature
- The speed of sound in a given environment

On the path along the nozzle surface marked as Line 2 in Figure 17, the following variables were plotted:

- Static pressure development
- Static temperature

**Figure 20:**
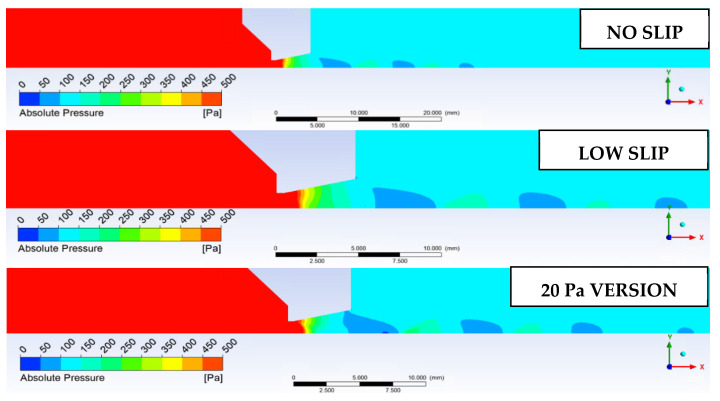
Distribution of Mach disks for the investigated variants.

These facts can be seen in Figure 20, where the distribution of Mach disks for the NO SLIP and LOW SLIP variants corresponds to the theory whereby the location of the first Mach disk corresponds to [21,22].

(5) $$z_{M}=0.67D_{kr}\sqrt{\frac{P_{0}}{P_{1}}}=5.95\;\mathrm{mm}$$

*D_kr_* is the critical cross-section dimension of 2 mm, *P*_0_ is the inlet static pressure, and P1 is the outlet static pressure.

With significantly reduced shear stress on the nozzle wall, the increased gas expansion results in a shorter distance. This fact will be tested together with the following methods using the Schlieren method of optical refraction [23].

**Figure 21:**
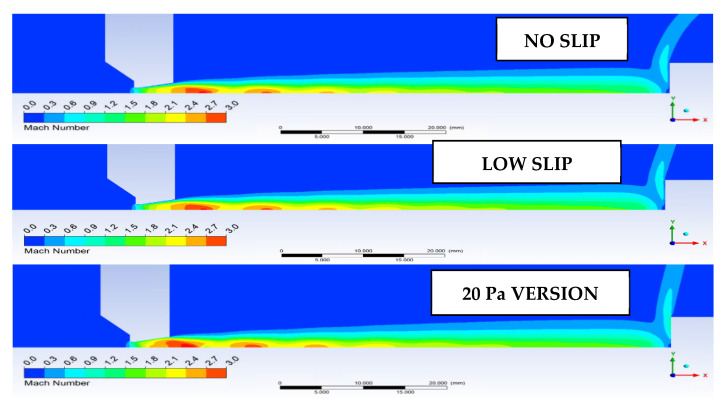
Mach number distribution.

These facts can be illustrated by the Mach number distribution in Figure 21.

These results are the basis for the upcoming experimental measurements, where the static pressure and Mach number will be measured on this path using Pitot tubes. These results will be one of the bases for the evaluation of the flow character, thereby allowing, among other things, the evaluation of the shear stress in the nozzle.

**Figure 22:**
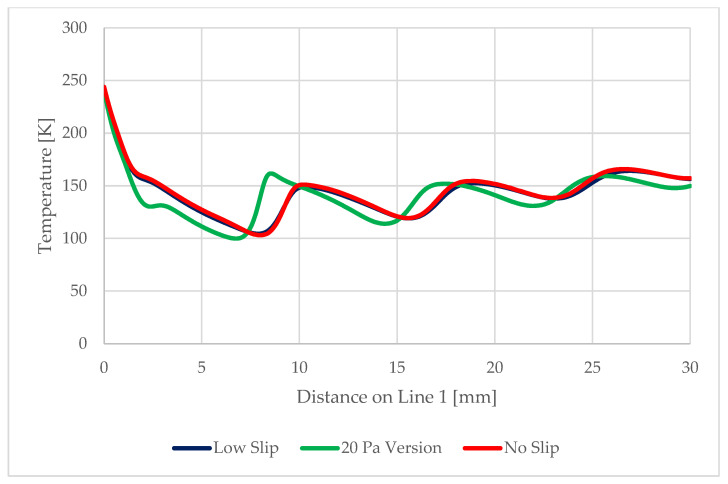
Static temperature evolution for the tested variants.

**Figure 23:**
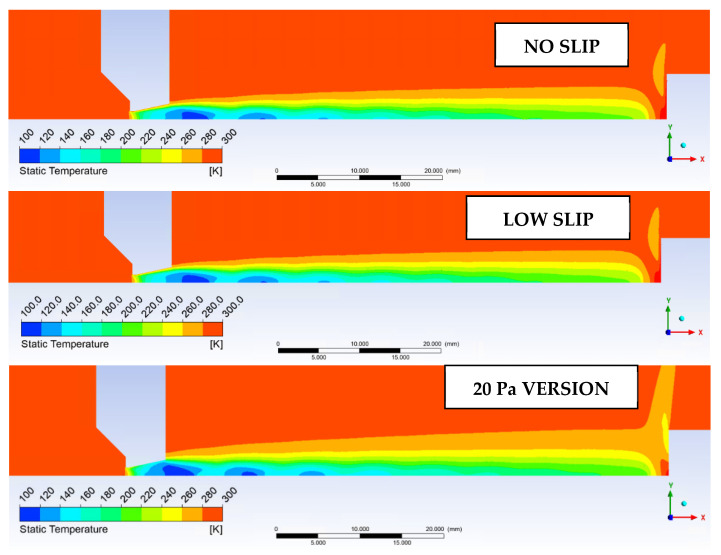
Static temperature distribution for the variants studied.

As seen in Figure 22 and Figure 23, next variable to be investigated will be the static temperature evolution on Line 1. This will be a verification of the measured results of static pressure and Mach number, given that these variables are closely related.

**Figure 24:**
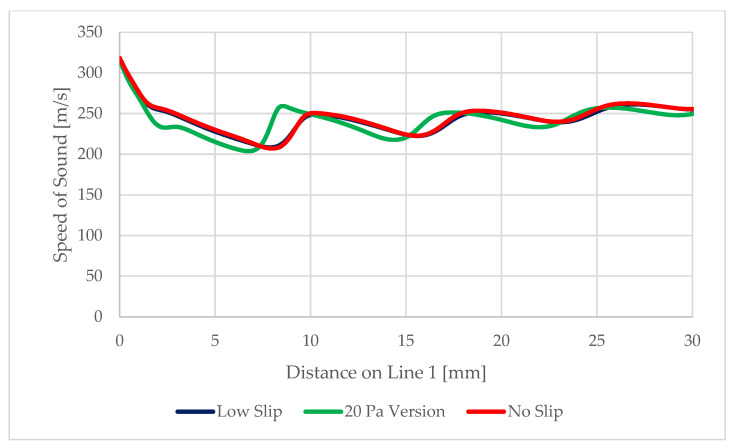
Speed of sound on Line 1.

**Figure 25:**
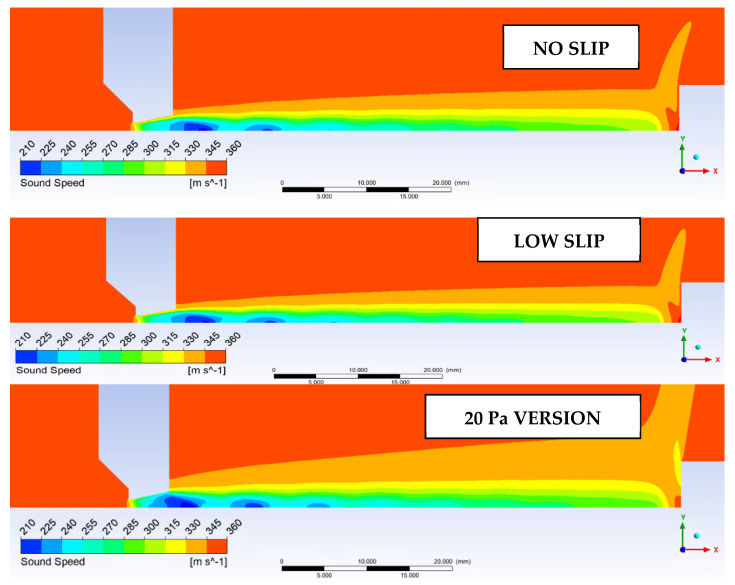
Distribution of the speed of sound in the experimental chamber.

Since we are considering pressure values and steep gradients in an extreme environment, it is a good idea to evaluate the speed of sound in that environment. The speed of sound at Line 1 is shown in Figure 24 and its distribution is in Figure 25.

**Figure 26:**
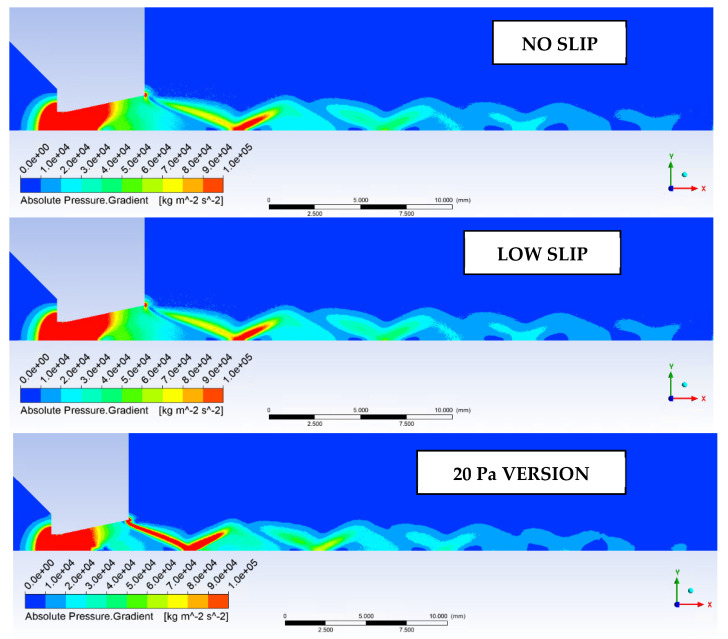
Pressure gradient distribution in the experimental chamber.

Another mapped variable, which will be observed by the Schlieren optical method, is the distribution of shock waves. This is based on the pressure gradient values shown in Figure 26. Here, it can be seen that due to the more pronounced expansion of the gas, the 20 Pa version has a very pronounced form of oblique shock waves emanating directly from the nozzle edge. This is because the rapid expansion is already occurring inside the nozzle, as indicated by the previous distribution of static pressure, flow velocity, and static temperature.

So far, results on the flow axis have been presented. Now, the results taken on the nozzle wall will be shown.

**Figure 27:**
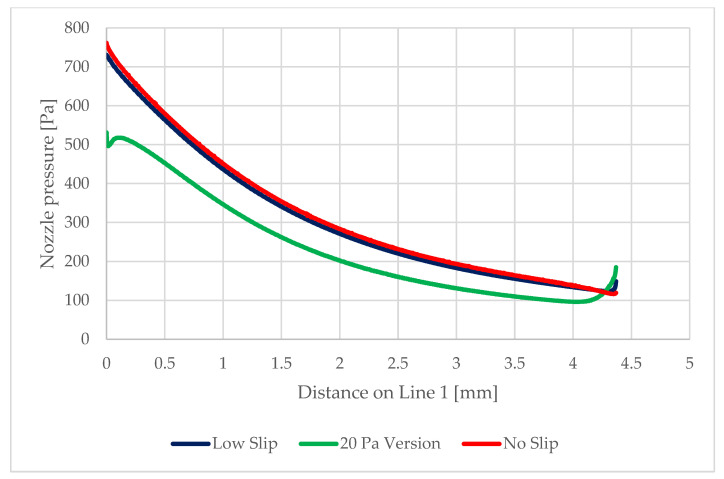
Static pressure distribution on the nozzle wall in the experimental chamber.

**Figure 28:**
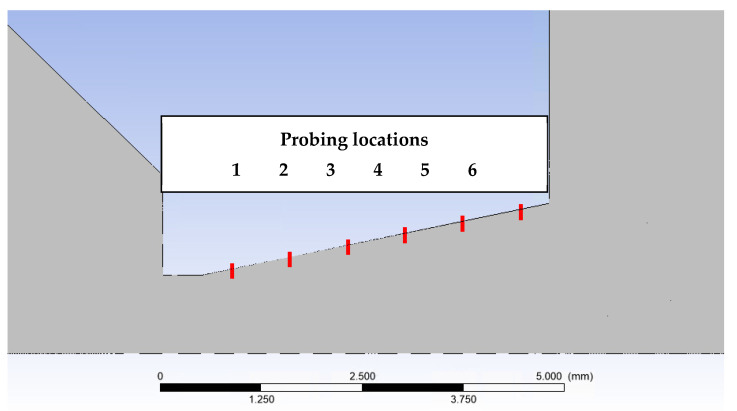
Distribution of reading points in the nozzle.

**Figure 29:**
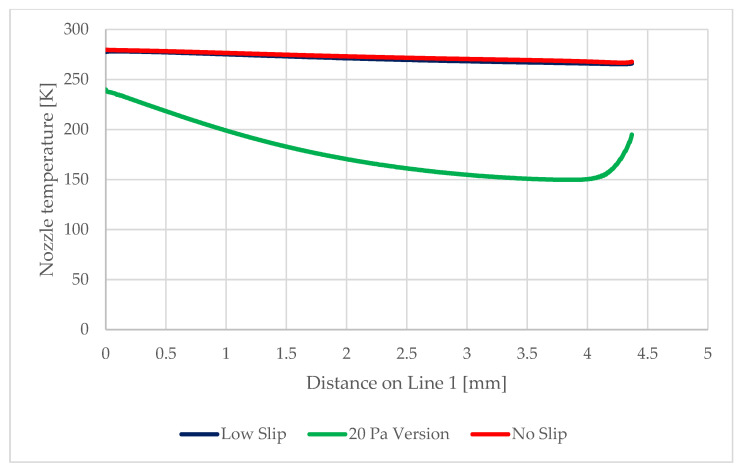
Temperature distribution on the nozzle wall in the experimental chamber.

Figure 27 shows the distribution of pressure profiles on the nozzle wall obtained from the mathematical and physical analyses in the Ansys Fluent. These analyses will be the basis for the debugging of the experimental measurement of these pressure ratios using static pressure reading probes. The assumed distribution of these probes for the experimental measurements is along the nozzle wall in spirally placed holes (Figure 28). Similarly, Figure 29 shows the distribution of temperature profiles on the nozzle wall obtained from mathematical and physical analyses.

From the results obtained from the mathematical–physical analyzes, it once again turns out that the difference between the no slip and low-pressure boundary slip versions is relatively small, although this difference already demonstrates the existence of a slip on the lower pressure and thus temperature path. This slip is already more significant for the version with a significantly reduced shear stress value. These values will be mapped experimentally in a given chamber and will provide further support to the measurements already mentioned.

The exact values at the sensed locations are shown in Table 3.

Finally, a basic check of the results of the mathematical–physical analysis was performed with the theory of one-dimensional isentropic flow in the region of Mach number and temperature progression.

For the isentropic flow applies [24]:

(6) $$\frac{T_{v}}{T_{o}}=\frac{2}{2+\left(\varkappa -1\right)M^{2}}$$

(7) $$\frac{p_{v}}{p_{o}}={\left[\frac{2}{2+\left(\varkappa -1\right)M^{2}}\right]}^{\frac{\varkappa }{\varkappa -1}}\;$$

where:

*T*_0_—input temperature, *T_v_*—output temperature, *p*_0_—input pressure, *p_v_*—output pressure, *M*—Mach number, ϰ—gas constant = 1.14.

From the pressure ratios (7), which were given as *p*_0_ = 2000 Pa and *p_v_* = 100 Pa, and from (6), the Mach number at the nozzle outlet cross-section is determined as 2.6, which corresponds to reality. Similarly, from (6) for the ratio of inlet temperature to the nozzle outlet temperature, a value of 127 K also agrees with reality.

## 6. Conclusions

The paper is a follow-up to the comparative analyses carried out at the Institute of Scientific Instruments of the CAS in cooperation with the Department of Electrical and Electronic Technology of the Brno University of Technology, using the Ansys Fluent system using continuum mechanics. Based on these analyses, an experimental chamber simulating the differential pumping condition in ESEM was constructed, in which it will be possible, among other things, to evaluate the slip flow on the nozzle walls. The experimental measurements were preceded by a series of analyses comparing the flow on the nozzle wall in several variants. Based on the implemented design of the experimental chamber for supersonic flow at low pressures at the limit of continuum mechanics, analyses were performed of the predicted Slip Flow conditions at the nozzle wall, where the conditions are at the limit of No Slip and Slip Flow. The analyses performed indicated the predicted pressure and temperature distributions at the nozzle wall. These results will be compared with the values obtained by the experiment, and the mathematical and physical model setup in Ansys will be adjusted. This comparison will be made after the subsequent tuning of the mathematical–physical model in the Ansys Fluent, namely with the pressure and temperature on the nozzle wall, the sensing of static, and the total pressure and temperature from the nozzle centerline area during the supersonic flow. Using this combination of experimental measurements and the mathematical–physical model, the actual Slip Flow at the nozzle wall under different pressure gradient conditions at the interface of continuum mechanics will be determined. These results of the combination of experimental measurements and mathematical–physical analyses will be further used for aperture design in ESEM microscopes containing differential pumping. Without the information obtained from these analyses, several time- and cost-consuming experiments would be required.

## Figures and Tables

**Table 1:** Comparison of values obtained by experimental measurements with results obtained by Ansys Fluent analyses.

Pressure in Specimen Chambre [Pa]	Pressure at the Probe in the Differentially Pumped Chamber–Experiment [Pa]	Pressure at the Probe in the Differentially Pumped Chamber–Ansys [Pa]
2300	16.2	16.19
2200	15.8	15.81
2100	15.2	15.22
1450	14.7	14.7
1400	14.3	14.31
1350	14	14.06
1300	13.8	13.81
1250	13.4	13.37
1200	13.2	13.2
1150	12.9	12.89
1100	12.7	12.71
1050	12.3	12.28
1000	12.1	12.02
950	11.7	11.74
900	11.5	11.46
850	11.2	11.22
800	10.9	10.84
750	10.6	10.61
700	10.3	10.29
650	10	9.95
600	9.7	9.71
550	9.4	9.35

**Table 2:** Values of monitored variables during chamber pumping.

Time [s]	Chamber Values		Nozzle Wall Values			
	P1	P2	Shear Stress [Pa]	Shear Stress x [Pa]	Shear Stress y [Pa]	P Nozzle [Pa]
0.1	80,578	17,120	155.4	134.1	30.1	30,831.5
0.2	60,554	7852	152.8	134.6	29.5	17,907.3
0.3	45,302	3600	140.2	126.2	27.1	11,006.6
0.4	33,957	1651	126.3	116	24.6	7076.5
0.5	25,302	1261.7	116.5	108.6	22.8	3564.9
0.6	18,886.9	943.9	104.5	97.7	20.4	2474.5
0.7	14,054.2	705	92	86.6	18	1739.4
0.8	10,487.3	558.7	76.7	72.7	15	1225.6
0.9	7893	437	65.3	62.6	12.7	903.8
1.0	5805	267.8	60.6	58.9	11.8	667.2
1.1	4290.8	221.4	54.7	53.5	10.6	507.5
1.2	3159	156	49.9	48.9	9.6	386.7
1.3	2332.5	118.3	45.2	44.3	8.6	298.7
1.4	1736.8	90.17	40.8	40	7.7	232.1
1.5	1308	69.2	36.4	35.7	6.8	184.8

**Table 3:** Values of variables at probing points.

	No-Slip		Low-PBS		20 Pa Version	
	Pressure [Pa]	Temperature [K]	Pressure [Pa]	Temperature [K]	Pressure [Pa]	Temperature [K]
Probe 1	620.2	278.7	603.4	277.7	481.3	224
Probe 2	431.1	276.3	416.2	274.9	328.5	195.7
Probe 3	306.4	273.8	293.6	272	221.2	174.5
Probe 4	227.1	271.6	215.5	269.6	156.1	160.2
Probe 5	176.3	270	166.6	267.7	118.3	152.2
Probe 6	137.3	268	133.6	266.1	96.4	150.5

## Data Availability

The data presented in this study are available on request from the corresponding author.
